# The key to intestinal health: a review and perspective on food additives

**DOI:** 10.3389/fnut.2024.1420358

**Published:** 2024-09-18

**Authors:** Haitao Wang, Junyi Bai, Pengyu Miao, Yu Wei, Xiaochao Chen, Haibo Lan, Yong Qing, Meizhu Zhao, Yanyu Li, Rui Tang, Xiangdong Yang

**Affiliations:** ^1^The School of Clinical Medical Sciences, Southwest Medical University, Luzhou, Sichuan, China; ^2^Chengdu Anorectal Hospital, Chengdu, Sichuan, China; ^3^School of Basic Medical Sciences, Southwest Medical University, Luzhou, Sichuan, China

**Keywords:** food additives, intestinal health, intestinal microecology, intestinal diseases, food safety regulation

## Abstract

In this review, we explore the effects of food additives on intestinal health. Food additives, such as preservatives, antioxidants and colorants, are widely used to improve food quality and extend shelf life. However, their effects on intestinal microecology May pose health risks. Starting from the basic functions of food additives and the importance of intestinal microecology, we analyze in detail how additives affect the diversity of intestinal flora, oxidative stress and immune responses. Additionally, we examine the association between food additives and intestinal disorders, including inflammatory bowel disease and irritable bowel syndrome, and how the timing, dosage, and individual differences affect the body’s response to additives. We also assess the safety and regulatory policies of food additives and explore the potential of natural additives. Finally, we propose future research directions, emphasizing the refinement of risk assessment methods and the creation of safer, innovative additives.

## 1 Introduction

Intestinal health is crucial for overall human well-being, acting as a cornerstone of physiological health (1). Food additives, used to enhance food quality, flavor, prevent microbial contamination, and extend shelf life, are increasingly utilized due to shifts in dietary patterns and advancements in food processing technologies (2). Food additives encompass a wide variety, including thickeners, colorants, sweeteners, emulsifiers, as well as flavors and seasonings (2). While the U.S. Food and Drug Administration (FDA) has deemed food additives relatively safe, many still pose potential health risks to humans (3, 4). For instance, non-caloric artificial sweeteners are among the most widely used food additives globally. Epidemiological studies and animal experiments suggest that long-term consumption of non-caloric sweeteners, such as aspartame, saccharin, and sucralose, can disrupt hormone levels, leading to impaired glucose tolerance in both experimental animals and healthy volunteers. These sweeteners also alter the expression of reward-related genes in the brains of offspring, exacerbating obesity and impaired glucose tolerance during adolescence (5).

The widespread use of food additives has raised health concerns, particularly regarding their effects on the gastrointestinal tract (4). Although early studies have identified some links between food additives and intestinal diseases, their findings are often fragmented and fail to offer a comprehensive view of how these additives affect intestinal microecology, oxidative stress, and immune responses. Moreover, research is limited on how various types, dosages, and exposure times of food additives affect intestinal health, and how individual differences May modulate these effects.

This paper aims to elucidate the complex relationships between food additives, intestinal flora, and intestinal health, with a particular focus on their links to intestinal diseases. Additionally, it assesses the safety of food additives and the role of food safety regulation in maintaining intestinal health, providing the public, researchers, and policymakers with a comprehensive perspective and deeper understanding of these relationships. It also aims to provide scientific evidence for the development of more effective public health policies and food safety standards, as well as to guide future research directions.

## 2 Food additives and intestinal microecology

### 2.1 Relationship between food additives and intestinal flora

The gastrointestinal (GI) tract hosts a diverse array of microbial populations (6). The intestinal microbial community consists of a diverse assembly of microorganisms—including bacteria, fungi, protozoa, and viruses—that colonize the intestinal tract (7). The major phyla of the intestinal microbiota include Bacteroidetes, Firmicutes, Clostridia, Proteobacteria, Cyanobacteria, Verrucomicrobia, and Actinobacteria (8). The healthy state of the intestinal microbiota significantly impacts overall health; however, food additives May alter the composition and function of the intestinal microbiota through various pathways. The interaction between food additives and intestinal microbiota is complex, exhibiting both beneficial and detrimental effects. In the following sections, we will describe the effects of various food additives on intestinal microbiota separately.

Preservatives, a prevalent category of food additives, possess antimicrobial properties designed to inhibit harmful bacteria, thereby extending the shelf life of food (9). Research indicates that certain antimicrobial preservatives can trigger abnormalities in glucose tolerance and interfere with the intestinal microbial community, negatively impacting intestinal health (10). Propionate, a type of preservative, can alter microbial composition and metabolism (11). Propionate is also a type of short-chain fatty acid (SCFA) that can modulate intestinal inflammation through immunomodulation and the regulation of the intestinal barrier (11). However, in patients with intestinal inflammation who are deficient in SCFAs, propionate supplementation can be beneficial (12).

Antioxidants play a critical role in preserving food freshness; however, they May also negatively impact the intestinal ecosystem. Polyphenols, a subgroup of antioxidants, are metabolized by intestinal microorganisms, thus influencing the diversity and activity of the intestinal microbiota (13). Studies have shown that polyphenols can alter the intestinal environment by modulating the metabolites of the intestinal microbiota, such as SCFAs, gasses, enzymes, etc., thereby affecting the composition and function of the microbiota. Research indicates that polyphenols May inhibit the growth of certain beneficial microorganisms, particularly lactic acid bacteria and some probiotics (14, 15), and that these inhibitory or bactericidal effects depend on the specific polyphenol structure and bacterial species. Indeed, preservatives can negatively impact the intestinal microbiota even when used within regulatory limits. For instance, certain food preservatives (e.g., benzoic acid, propionate) can reduce the diversity of the intestinal microbiota and induce mild intestinal inflammation (16).

Flavor enhancers, which include various substances, are used to improve the taste profiles of foods. The most commonly used flavor enhancer is monosodium glutamate (MSG), the sodium salt of glutamic acid (17). Xu et al. (18) investigated the intestinal structure and microbiota of mice orally administered MSG via tube feeding. The results indicated that moderate intake of MSG can increase the ratio of Bacteroidetes to Firmicutes and enhance the abundance of probiotic bacteria, promoting intestinal development and regulating the intestinal microbiota composition. However, excessive intake of MSG can reduce the abundance of certain microbiota, such as bifidobacteria (19). Conversely, Peng et al. (20) observed that MSG consumption did not significantly alter the structural or functional characteristics of the intestinal microbial community in the subjects, which May be attributed to the ingested amount or the physiological characteristics of the organism.

Artificial sweeteners, such as aspartame, acesulfame, sucralose, saccharin, neotame, and their derivatives, are commonly found in sugar-free beverages, confectioneries, and dairy products (21). Recent research has highlighted alterations in the intestinal microbiota and metabolites attributed to these sweeteners (22). Specifically, chronic low-dose aspartame consumption in mice has been shown to increase the populations of *Clostridium leptum* and Enterobacteriaceae (23). Additionally, aspartame enhances the adhesion, invasion, and epithelial lethality of *Escherichia coli* NCTC10418 and *Enterococcus faecalis* ATCC19433 (24). Studies by Palmnäs and Nettleton reported that aspartame intake elevates serum, fecal, and cecal concentrations of propionic and butyric acids, indicating alterations in the metabolic processing within the intestinal microbiota (5, 23). Acesulfame intake has demonstrated significant gender-specific effects, with females experiencing reduced levels of Lactobacillus and Clostridium but increased Mucispirillum, whereas males exhibited increased Bacteroides, Sutterella, and Anaerostipes (25). In pregnant and lactating mice, acesulfame intake was linked to increased levels of Bacillota and decreased levels of the potentially anti-inflammatory *Akkermansia muciniphila* (26). Contrasting studies on sucralose indicate no impact on intestinal microorganisms after short-term consumption (27). However, prolonged intake over 10 weeks in young individuals has been linked to increased *Blautia coccoides* and decreased *Lactobacillus acidophilus*, along with broader disturbances in intestinal flora and fluctuations in serum insulin and glucose levels (28). Elevated concentrations of saccharin in cecal contents are associated with increased aerobic flora (29), and exposure to saccharin has led to changes in intestinal microbiota composition and function, potentially promoting glucose intolerance (30). Saccharin also increases populations of Ackermannia, Corynebacterium, and Turicibacter, while decreasing Anaerostipes, Ruminococcus, and Dorea (31). However, it has been noted to inhibit bacterial growth and mitigate experimental colitis in mice (32). Lastly, neotame intake caused a sharp decline in Firmicutes and an increase in the abundance of Bacteroides, particularly Bacteroides, with significant reductions in various members of the Trichoderma and Ruminalococcaceae families, including Blautia, Dorea, Oscillospira, and Ruminalococcus spp (33).

Sugar alcohols are widely used in the production of chemicals, pharmaceuticals, and foods, including erythritol, xylitol, lactitol, and sorbitol (34). Erythritol has been found to increase intestinal metabolites such as butyric and valeric acids, though it does not significantly alter the structure of the intestinal microbiota (35). Conversely, xylitol May shift the rodent intestinal microbial population from Gram-negative to Gram-positive bacteria (36) and has been shown to decrease the relative abundance of Proteobacteria, Bacteroidetes, and Actinobacteria, while increasing the abundance of Bacillota and the ratio of Bacillota to Bacteroidetes in mice (37–39). Furthermore, Chen et al. (40) reported that lactitol intake significantly boosts the populations of bifidobacteria and lactobacilli while reducing the presence of *Clostridium perfringens* in patients with chronic viral hepatitis, noting that lactitol more effectively reduces plasma endotoxin levels than standard drug therapy. Other studies have shown that lactitol positively affects fecal flora and May alleviate symptoms such as constipation, thereby classifying it as a prebiotic (41, 42). Sorbitol is known to increase bacterial density and acetate synthesis (43). However, Li et al. (44) observed that chronic intake of sorbitol significantly decreased the relative abundance of various beneficial bacteria, including Bifidobacterium and several Lachnospiraceae members, while increasing the presence of pathogenic bacteria such as *H. pylori* and Prevotella, which subsequently induced glucose intolerance in mice.

Additionally, azo dyes commonly added to beverages like milk tea and ice cream, including lemon yellow, sunset yellow, and camosin (45), are metabolized by certain intestinal microorganisms such as Anabaena ovale and *Enterococcus faecalis*, which produce azoreductases. These enzymes convert azo dyes into the sodium salt of 1-amino-2-naphthol-6-sulfonate, a metabolite known to cause colitis (46). In their study, Wu et al. (47) used crucian carp (*Carassius auratus*) as an experimental model to assess the effects of lemon-yellow consumption. They demonstrated significant alterations in the intestinal microbial community of the fish, with a decrease in beneficial probiotics such as Roseomonas, Rhodococcus, and Bacillus, and an increase in pathogenic microorganisms like Vibrio vermiculiformis and Schizosaccharomyces cerevisiae, which negatively impacting fish health.

Emulsifiers, including carboxymethyl cellulose (CMC) and polysorbate 80 (P80), have been associated with a heightened prevalence of intestinal inflammation-related disorders, such as inflammatory bowel disease and metabolic syndrome. This association is attributed to the emulsifiers’ capacity to directly alter the intestinal microbiota, thereby promoting inflammation (48). Chassaing et al. (49) observed that healthy adults consuming a diet enriched with 15 grams per day of CMC exhibited changes in the fecal metabolome, notably a reduction in SCFAs and free amino acids, compared to a control group. Furthermore, two subjects in the CMC group demonstrated increased microbial invasion into the normally sterile inner mucus layer—a hallmark of intestinal inflammation—and significant shifts in microbiota composition (49). Moreover, carrageenan consumption was found to increase the presence of phyla such as Proteobacteria and Deferribacteres, while reducing Firmicutes, Actinobacteria and Bacteroidetes (50).

Conversely, not all food additives pose health risks. The International Scientific Association for Probiotics and Prebiotics (ISAPP) acknowledges prebiotics for their health-enhancing effects, emphasizing their selective use by the host’s intestinal flora to generate health-promoting metabolites such as SCFAs (51). These metabolites not only inhibit pathogen growth but also activate immune responses, helping to prevent infections and allergies (51, 52). As a prebiotic, galacto-oligosaccharides (GOS) have been shown to positively impact intestinal health, particularly in immunomodulation and the improvement of IBD. A high-purity GOS product, NeoGOS-P70, has been shown to effectively alleviate DSS-induced colitis in mice, reducing symptoms and colon shortening (53).

Ultimately, food additives exert a complex array of effects on intestinal microbiota, encompassing both potential risks and benefits. Prolonged exposure to specific food additives May disrupt microbiota structure and function, potentially leading to ecological imbalances and diseases such as ulcerative colitis and Crohn’s disease (54). Moreover, antimicrobial food additives May diminish sensitive bacterial populations while increasing others, thus altering the diversity and stability of the intestinal microbial community (55). These changes can impact the host’s immune system and overall health. However, the potential benefits of additives like propionate, monosodium glutamate, and lactitol for intestinal health cannot be overlooked. Future research ought to investigate further the precise effects of various food additives on intestinal flora and their implications for human health, with the goal of optimizing their use to enhance rather than undermine intestinal health (Table 1).

**Table 1:** Overview of the effects of different food additives on intestinal flora.

Types of food additives	Specific additives	Mechanism of action	Effects on microorganisms	Other effects
Preservative	Propionate (11, 12)	Altered microbial composition and metabolism	May inhibit beneficial microorganisms	May cause inflammation, but beneficial in patients with intestinal inflammation who lack SCFAs
Antioxidant	Polyphenol (14, 15)	Inhibit certain beneficial microorganisms	May inhibit lactic acid bacteria and certain probiotics	Affect intestinal environment and microbial composition
Flavor enhancer	Monosodium glutamate (MSG) (18, 19)	Increase abundance of certain probiotics	May inhibit bifidobacteria	Moderate amounts May have beneficial effects
Sweetener	Aspartame (5, 23), acesulfame (25, 26)	Alter intestinal microbiota and metabolites	Cause changes in intestinal microbiota	Affect intestinal health
Sugar alcohol	Erythritol (35), xylitol (36)	Alter intestinal microbiota and metabolites	Cause changes in intestinal microbiota	Limited effect on intestinal microbiota
Azo dye	Lemon yellow (47)	Alter structure of intestinal microbial community	May reduce the number of beneficial bacteria and increase the number of pathogenic bacteria	Can have a negative impact on intestinal health
Emulsifier	P80 (48), CMC (49)	Lead to changes in microbiota composition	May cause microbiota to invade the normally sterile inner mucus layer	Cause intestinal inflammation
Probiotic	Galacto-oligosaccharides (GOS) (53)	Promote beneficial metabolite production	Promote the production of beneficial metabolites by beneficial microorganisms	Improve intestinal health

### 2.2 Relationship between food additives and intestinal oxidative stress

Intestinal oxidative stress occurs when the production of oxidants exceeds the antioxidant scavenging capacity within the intestinal tract, potentially leading to pathologies such as inflammation and cancer (56). Food additives May exacerbate this condition by increasing oxidant levels or impairing antioxidant functions, consequently compromising intestinal barrier integrity.

To commence, direct interactions between food additives and intestinal cells can elevate levels of reactive oxygen species (ROS) and reactive nitrogen species (RNS). For example, fructose has been shown to promote intestinal permeability through ethanol-induced cytochrome P450-2E1-mediated oxidative and nitrative stress, increasing inducible nitric oxide synthase (iNOS) in rat plasma, thereby exacerbating oxidative stress (57). Prolonged exposure to titanium dioxide (E171) induces oxidative stress and DNA base oxidation in intestinal epithelial cells (58). Additionally, diets rich in maltodextrin (MDX) disrupt the intestinal mucus barrier, induce endoplasmic reticulum stress, and heighten inflammatory responses in these cells (59). Dietary emulsifiers May also indirectly increase oxidative stress by diminishing the protective mucosal layer and promoting the growth of pro-inflammatory microbiota (60).

Subsequently, oxidative stress damages intestinal cells and disrupts mucosal barrier integrity, enhancing permeability and allowing harmful antigens to penetrate, which leads to systemic inflammation (61). Piglets on a fructose-rich diet showed reduced tight junction gene expression and altered microbiota, leading to barrier dysfunction and inflammation (62). Research also suggests that high concentrations of artificial sweeteners, such as aspartame and saccharin, can trigger apoptosis and enhance epithelial barrier permeability, disturbing tight junctions and fostering ROS production (63).

Furthermore, some food additives impair intestinal antioxidant defenses. For instance, lemon yellow exposure in crucian carp has been linked to reduced activity of key antioxidant enzymes like CAT, SOD, and GSH-Px, thereby promoting oxidative stress (64). Conversely, in a mouse model of colitis, Pingyin rose essential oil (PREO) was found to enhance the enzymatic activities of SOD and catalase (CAT), while reducing NO, malondialdehyde (MDA) and myeloperoxidase (MPO) production and restoring intestinal barrier integrity (65).

**Figure 1:**
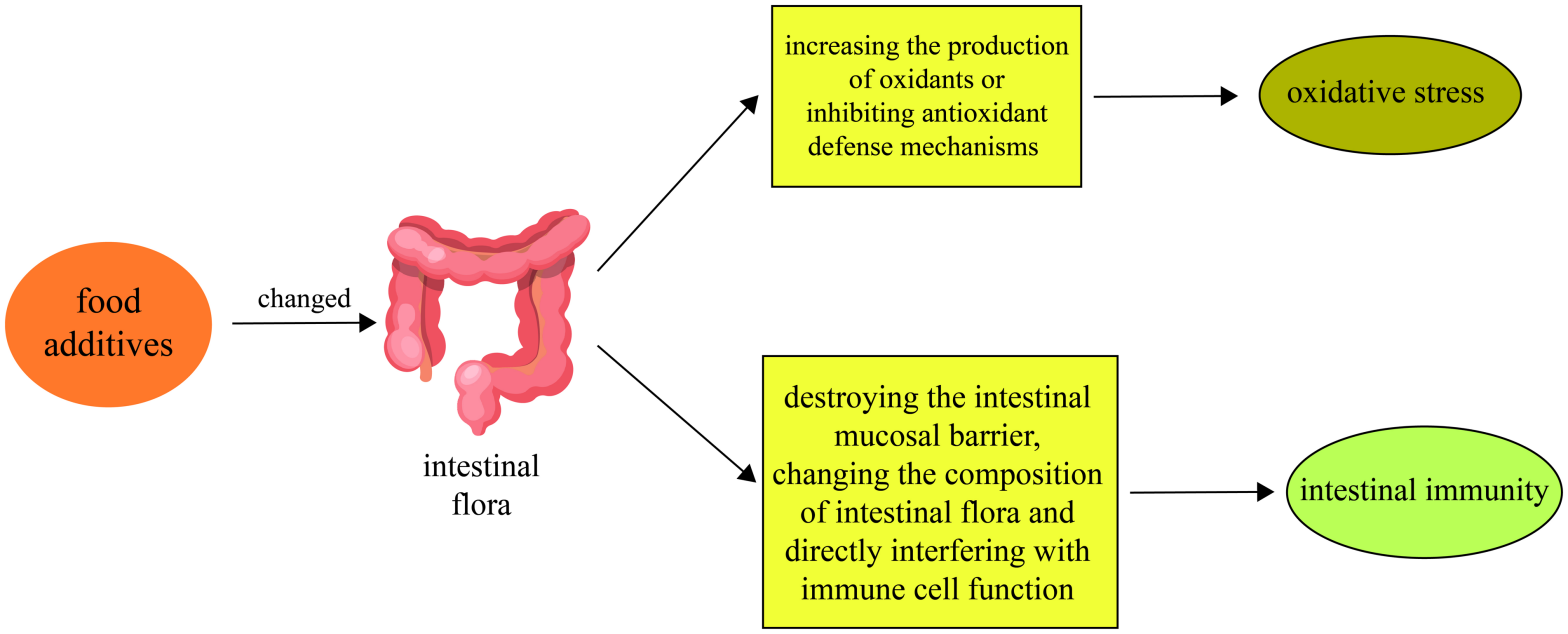
Food additives alter physiological mechanisms such as oxidative stress and intestinal immunity by affecting intestinal microbiota. First, food additives can change the composition and metabolism of the intestinal flora. Second, food additives May exacerbate intestinal oxidative stress by increasing the production of oxidants or inhibiting antioxidant defense mechanisms. Finally, food additives can also affect the intestinal immune response by disrupting the intestinal mucosal barrier, changing the composition of the intestinal flora, and directly interfering with immune cell function.

Overall, the impacts of food additives on intestinal oxidative stress are diverse, encompassing direct boosts in oxidant production, disturbance of intestinal barrier function, and impairment of intestinal antioxidant defense mechanisms. The combined effect of these actions May compromise intestinal health and increase the risk of chronic intestinal diseases. Therefore, understanding how food additives influence intestinal oxidative stress mechanisms is crucial for assessing and managing the safety of these additives (Figure 1).

### 2.3 Relationship between food additives and intestinal immunity

The intestinal tract, the body’s largest immune organ, plays a critical role in maintaining the balance of the intestinal microbial community and safeguarding against harmful microorganisms (66). Diet substantially influences this balance and directly affects intestinal and immune homeostasis (67). Hence, understanding the mechanisms by which food additives affect intestinal immune responses is vital for assessing their safety. Food additives influence the intestinal immune system by disrupting the mucosal barrier, altering microbial composition, and modulating immune cell responses.

To commence, food additives compromise the intestinal mucosal barrier, the primary defense against pathogens and allergens (68). Chassaing et al. (60) showed that emulsifiers enhance the abundance of Ruminicoccus gnavus and *Akkermansia muciniphila*, while thinning the mucus layer, alterations that correspond with heightened intestinal permeability and increased levels of lipopolysaccharide (LPS) and circulating flagellin in mice. LPS is a major bacterial endotoxin tencountered by the immune system, and flagellin is a potent immune activator that triggers responses during infections (69). Additionally, TiO2 nanoparticles in additives are linked to inflammatory intestinal damage, which attributed to diminished mucus barrier function and elevated metabolite lipopolysaccharides, activating inflammatory pathways (70).

Subsequently, the intestinal microbial community, essential for both human immunity and intestinal health, can be significantly altered by food additives (71). Research has indicated that polyphenols can inhibit the growth of beneficial microorganisms, especially lactic acid bacteria and specific probiotics, with the impact contingent on the polyphenols’ structure and the bacterial species (14, 15). Furthermore, Li et al. (44) found that long-term sorbitol consumption markedly decreased the abundance of beneficial bacteria such as *Bifidobacterium* and increased levels of pathogenic bacteria such as *H. pylori*, leading to glucose intolerance in mice.

Furthermore, beyond affecting the intestinal barrier and microbial composition, food additives directly interfere with immune cell function. For example, high salt intake alters helper T cell function and promotes inflammation, potentially contributing to conditions such hypertension and obesity (72). High doses of titanium dioxide (TiO2) increase the production of inflammatory cytokines like IL-8, TNF-*α*, and IL-10 by macrophages, compromising their phagocytic activity, and disturbing the balance of dendritic and regulatory T cells (73, 74). Moreover, high fructose corn syrup (HFCS) disrupts intestinal flora, leading to a decrease in secondary bile acids and an imbalance in Th17/Treg cells, which can exacerbate intestinal inflammation (75) (Figure 1).

## 3 Food additives and intestinal health

### 3.1 Relationship between food additives and intestinal diseases

Firstly, food additives have been implicated in the escalation of the risk of inflammatory bowel disease (IBD). IBD is a chronic, progressive, immune-mediated inflammatory disease of the gastrointestinal tract characterized by complex pathogenesis involving environmental factors, homeostatic dysregulation, oxidative stress, and immune dysregulation (76). Food additives May increase the risk of IBD by influencing these factors. For instance, a high salt diet (HSD) was shown to exacerbate colitis in an IL-10-deficient mouse model and infectious colitis scenarios (77). This effect is attributed to HSD’s enhancement of pro-inflammatory cytokine expression, thereby identifying high dietary salt intake as a critical environmental factor in IBD inflammation (77). Aguiar et al. (78) also demonstrated that dietary salt causes intestinal inflammation, potentially by increasing intestinal permeability. Moreover, maltodextrin consumption has been linked to intestinal dysbiosis and heightened disease susceptibility, a recent study found that maltodextrin in drinking water exacerbated the inflammatory response in an intestinal disease model through mechanisms involving endoplasmic reticulum stress and mucin depletion (59). Carrageenan, a polysaccharide commonly used as a food additive, has been associated with increased inflammatory indices in patients with chronic intake, such as elevated IL-6 and fecal calreticulin (79). Dietary emulsifiers have been shown to alter intestinal microbiota composition, promote intestinal hyperpermeability, and increase inflammatory markers such as fecal lipid transport protein-2 in IL-10-deficient mice (60).

Furthermore, food additives contribute to the heightened risk of intestinal infections. TiO2, commonly used as a whitening agent in various food products, has been observed to exacerbate IBD and contribute to the development of intestinal infections in animal studies (80). The stability of the intestinal microbial community is crucial for resisting infections, and food additives can disrupt this balance, heightening infection risk (81). Benzoic acid, widely used as an antimicrobial preservative in food and feed (82), has shown benefits for growth and health by promoting intestinal functions such as digestion, absorption, and barrier function (83). However, overexposure to benzoic acid can result in toxicity and serious health and growth impairments in humans and animals, affecting organs like the liver, spleen, and lungs, and altering the intestinal mucosa, thereby increasing infection risks (84).

In addition, food additives further amplify the risk of intestinal tumors. The development of intestinal tumors, including colorectal cancer (CRC), is closely linked to factors such as intestinal flora imbalance, oxidative stress, and immune abnormalities (85, 86). Dysbiosis of intestinal flora, promoted by food additives, can enhance CRC progression through mechanisms involving inflammation, pathogenic bacteria and their virulence factors, oxidative stress, bacterial metabolites, and biofilms (87). Emulsifiers May foster intestinal and metabolic diseases by disrupting microbial community structures, impairing mucosal barriers, facilitating bacterial translocation, and triggering inflammation. These processes contribute to immune imbalances and elevated levels of circulating bacteria, significantly increasing the risk of neoplastic diseases (88). Regular consumption of dietary emulsifiers like carboxymethylcellulose or polysorbate-80 has been shown to induce microbiome alterations and low-grade inflammation that promote colon carcinogenesis (89). Additionally, emulsifiers exacerbate the development of spontaneous intestinal adenomas by affecting the proliferative state of the intestinal epithelium (90). Triclosan (TCS), an antimicrobial additive, modulates intestinal microbiota and TLR4 signaling, increasing colonic inflammation and colitis-associated colon tumorigenesis in mice (91).

**Figure 2:**
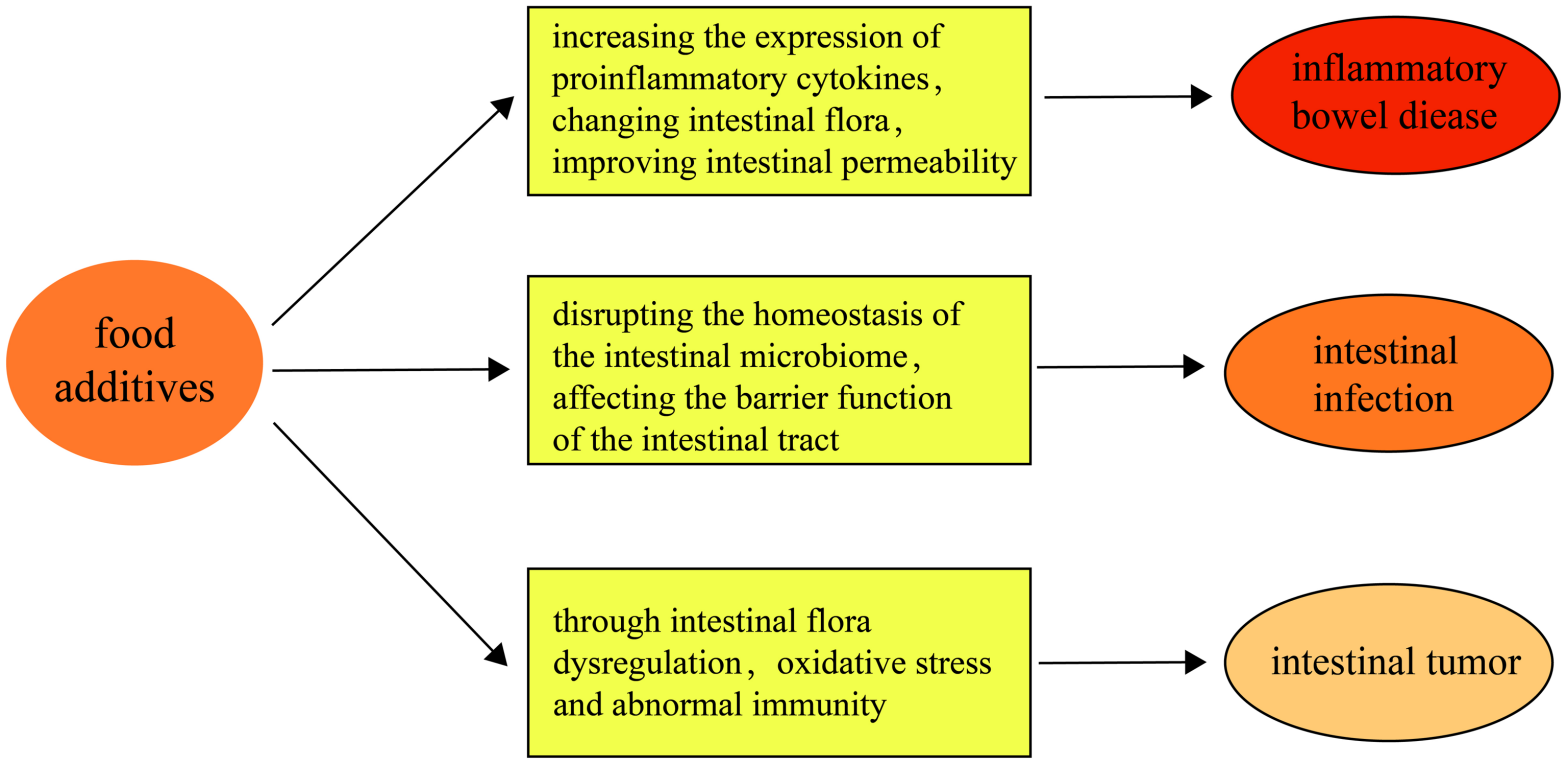
First of all, food additives aggravate the occurrence of inflammatory bowel disease by increasing the expression of pro-inflammatory cytokines, changing intestinal flora, improving intestinal permeability and other mechanisms. Secondly, food additives May impair the barrier function of the intestinal tract by disrupting the homeostasis of the intestinal microbiome, thereby increasing the risk of intestinal infections. Finally, food additives May increase the risk of intestinal tumors through the pathway of intestinal flora imbalance, oxidative stress and abnormal immunity.

Summarizing, the connection between food additives and intestinal diseases entails a multifaceted interplay among intestinal flora dysbiosis, compromised barrier function, and inflammation. While the convenience of processed foods is significant, their consumption poses potential risks to intestinal health. Moving forward, future research must concentrate on unraveling the mechanisms through which food additives impact intestinal disease development and on formulating dietary guidelines aimed at reducing these risks (Figure 2).

### 3.2 Effect of type, dose and exposure time of food additives on intestinal diseases

The impact of food additives on intestinal diseases is determined by their type, dose, and duration of exposure. This section explores these factors in detail.

Initially, various types of food additives have been linked to different intestinal diseases. Dietary emulsifiers, for instance, May exacerbate inflammatory bowel disease (IBD) by promoting the growth of pro-inflammatory intestinal microbiota, disrupting intestinal mucus structure, increasing intestinal permeability, activating inflammatory pathways, and disrupting the cell cycle (4). Sweeteners like high fructose corn syrup (HFCS) are known to enhance intestinal tumor growth in mice, while aspartame and saccharin May elevate the incidence and recurrence of Crohn’s disease by affecting the growth of beneficial bacteria (92, 93). Food preservatives such as sulfites can induce an imbalance in biothiol levels in NCM460 colon cells, triggering intestinal inflammation in mice (94). Chronic exposure to synthetic food colorants, like Allura Red AC, exacerbates colitis models in mice through colonic 5-HT in both intestinal flora-dependent and independent pathways (95). Thickeners, such as carrageenan, have also been associated with inflammatory bowel disease (96), and xanthan gum-containing thickeners have been linked to necrotizing small bowel colitis in infants (97).

Subsequently, the dosage of food additives significantly influences intestinal disorders. For example, a study evaluating different doses of MSG on intestinal function in mice set up groups at 30 mg/kg, 300 mg/kg, and 1,500 mg/kg. The study found that a lower dose (30 mg/kg) promoted the growth of intestinal villi and enhanced absorption functions, whereas a high dose (1,500 mg/kg) decreased the villi-to-crypt ratio and increased albumin leakage, indicating impaired intestinal barrier function (18). Additionally, Yang et al. (98) found that sorbitol concentrations were significantly higher in the feces of patients with active IBD compared to those in remission or healthy controls. Higher daily consumption of lemon yellow in carp correlated with more severe histopathological changes in the intestinal tract and increased pro-inflammatory cytokines (47). Katsoudas et al.’s analysis (99) revealed that total emulsifier exposure was significantly higher in patients with inflammatory bowel disease than in healthy controls.

Furthermore, the duration of exposure to food additives also affects intestinal health. Xu et al. (100) observed that by day 120, nitrite exposure in C57BL/6 mice led to a significant increase in alpha-diversity, as indicated by higher Chao 1 and Shannon indices, and a greater number of distinct genera compared to day 70. Chronic nitrite exposure decreased the abundance of Elusimicrobium and Akkermansia, microorganisms linked to reduced obesity and inflammation (101).

In conclusion, the type, dose, and duration of exposure to food additives collectively influence intestinal health. This underscores the need to consider these factors in research, along with individual differences such as genetic background, dietary habits, and lifestyle, which can influence responses to food additives.

### 3.3 Individual differences in food additives and intestinal diseases

Individual differences play a crucial role in understanding the interaction between food additives and intestinal diseases. This underscores the need to consider these factors in research, along with individual differences such as genetic background, dietary habits, and lifestyle, which can influence responses to food additives.

Firstly, genetic factors are pivotal in determining individual responses to food additives. Specific genetic variants, such as single nucleotide polymorphisms (SNPs), significantly influence the composition and function of intestinal microbes (102), which, in turn, affect the metabolism of food additives and their impact on intestinal health. For instance, the presence of the bacterium *Akkermansia muciniphila* has been shown to mitigate the harmful effects of dietary emulsifiers on the microbiota and host metabolism, preventing low-grade inflammatory responses and metabolic dysregulation, thus supporting intestinal health (103). Zou et al. (104) highlighted the protective role of intestinal bacteria in mitigating the adverse effects of food and drug additives on the intestinal tract.

Subsequently, lifestyle variations, particularly in terms of exercise habits, sleep patterns, and dietary habits, profoundly influence the composition of intestinal flora, which, in turn, affects an individual’s response to food additives. For example, exercise-focused dietary strategies such as protein supplementation and carbohydrate loading have been shown to benefit the intestinal microbiota of athletes and enhance intestinal barrier function (105). Moderate exercise also promotes beneficial changes in the composition of the intestinal microbiota and the microbial metabolites produced in the gastrointestinal tract, potentially reducing intestinal permeability (106). Voigt et al. (107) reported that disturbances in circadian rhythm are associated with dysbiosis of the intestinal flora. Thus, maintaining a consistent sleep and rest routine is vital for preserving the homeostasis of the intestinal flora, enhancing resistance to the negative impacts of food additives. Moreover, food composition and dietary habits are key determinants of the intestinal microbial community (108). A study indicated that deprivation of dietary fiber compromises the colonic mucus barrier and disrupts intestinal barrier function in mice (109).

In conclusion, individual differences in response to food additives and their impact on intestinal health are influenced by genetics, lifestyle, and dietary habits. Future research should further investigate how these factors contribute to individual variances and explore strategies to mitigate the adverse effects of food additives on intestinal health.

## 4 Food additive safety, alternative options and food safety regulation

### 4.1 Safety and risk assessment of food additives

Safety and risk assessment are foundational in the research and application of food additives. This section aims to provide an overview of the current methodologies in safety assessment and recent research findings, offering a scientific foundation for food safety management.

The safety assessment process for food additives typically includes identifying and quantifying potentially hazardous components, evaluating human exposure levels, and conducting risk analyses based on toxicological data (110). This multifaceted process integrates various factors such as the type of food additive, dosage, exposure duration, and individual differences. Advanced technologies enable rapid screening of additives in food products (111), and comprehensive databases of food ingredients have been developed to facilitate this process (112).

Recent risk assessment outcomes indicate that many food additives are safe at current usage levels. However, a meta-analysis identified that some artificial sweeteners May increase cancer risk in European populations (113). Accordingly, it is recommended that further long-term safety studies be conducted on such additives, and usage guidelines be revised as necessary.

The global food supply chain’s complexity and the diversifying types of food additives pose increasing challenges to safety assessments. International food safety organizations, such as the Joint Expert Committee on Food Additives (JECFA) of the World Health Organization (WHO) and the Food and Agriculture Organization (FAO), play critical roles in setting global food safety standards (114). Looking ahead, enhancing safety assessments for food additives will require strengthened international collaboration, greater transparency, and increased public involvement in risk assessments. Moreover, leveraging emerging technologies to improve the accuracy and efficiency of these assessments will be crucial.

### 4.2 Alternatives to food additives and natural additives

In response to the potential risks associated with synthetic food additives, researchers are actively exploring alternatives, including natural additives. Promoting and applying these safer alternatives supports public intestinal health protection.

Natural colorants are substances derived from natural plants, commonly including anthocyanins and betaines (115, 116). These colorants not only possess coloring properties but also exhibit various biological activities beneficial to health (115). Anthocyanins, found in the flowers and fruits of many plants, color most red, purple, and blue botanicals (117). Beyond coloring, anthocyanin-rich plants are utilized in treating various diseases due to their anti-inflammatory, anticancer, and cardioprotective properties. Additionally, anthocyanins help regulate cholesterol, LDL, HDL, triglycerides, and mitigate neurological and cognitive alterations (118). Betaine, sourced from beets, spinach, and grains, displays antioxidant, antimicrobial, and anticancer properties (119). Its antioxidant capability is linked to its chemical structure, containing phenolic groups and cyclic amines, which effectively combat free radicals (120). This highlights natural colorants’ superior safety and nutritional value.

Traditional preservatives, some of which can be chemically hazardous, are being replaced due to potential adverse effects when intake exceeds safe limits (121). As food safety demands increase, the industry is shifting toward natural preservatives, including antimicrobials and antioxidants derived from natural sources (122). Natural antimicrobials, secondary metabolites from plants (herbs, spices), animals (eggs, milk), and microorganisms (fungi, bacteria), exhibit antimicrobial properties (123, 124) and are increasingly used in food applications due to their disease-control effects (125). Common natural antioxidants like polyphenols, vitamins, and carotenoids, known for their potent antioxidant capacity (126), are employed in treating aging, cancer, diabetes, inflammation, liver disease, cardiovascular issues, and neurodegenerative diseases due to their favorable safety profiles (127).

Despite the benefits, natural additives face significant application challenges. Extracting and maintaining the stability and activity of these ingredients, especially on a large scale, poses major technical and cost challenges. Additionally, the viability and efficacy of certain natural additives, such as probiotics, May be limited in certain conditions, necessitating further research to optimize their formulation and application conditions. Despite these hurdles, the development and application of natural additives hold substantial potential. Future research should focus on enhancing the stability, efficacy, and cost-effectiveness of these additives, ensuring their suitability across various food systems. Further clinical and epidemiological studies are crucial to fully assess the long-term impacts of natural additives on human health, particularly regarding intestinal health.

### 4.3 The role of food safety regulation in preventing enteric diseases

Unhealthy diets and diet-related non-communicable diseases (NCDs) can increase societal costs indirectly and directly, placing a significant burden on healthcare systems and society at large (128). Therefore, government bodies must strengthen the regulation of food production, processing, and distribution, and strictly monitor the use of food additives to ensure food safety and hygiene.

The essence of regulatory policies is the safety assessment of food additives, which involves evaluating their potential toxicity, estimating exposure, and conducting risk assessments for specific populations, such as children and pregnant women (129, 130). In recent years, there has been a heightened focus on the effects of food additives on intestinal health. Numerous regulatory policies and laws have been enacted to improve food regulatory frameworks to minimize the impact of food additives on both intestinal and systemic health (131). Moreover, preventing intestinal diseases, including food poisoning and intestinal infections, is a crucial aspect of food safety regulation (132). These diseases are often closely linked to food contamination and the misuse of food additives. Strengthening the training and management of food service employees to enhance their food safety awareness and operational skills can help prevent the occurrence of enteric diseases at the source.

Despite strict regulatory policies, numerous challenges persist in practice. The complexity of the global food supply chain necessitates greater international cooperation and information sharing to effectively regulate food additives. Additionally, as new food technologies and novel additives rapidly develop, regulatory frameworks must continually evolve to accommodate these changes. Future regulatory policy development will require more evidence-based research, particularly in evaluating the long-term effects of food additives on intestinal health. The integration of emerging technologies, such as high-throughput screening and artificial intelligence, could enhance the efficiency and accuracy of food additive assessments. Moreover, enhancing consumer education and raising public awareness about the safety of food additives is a vital component of future regulatory strategies.

## 5 Conclusions and outlook

In this review, we examined the influence of food additives on intestinal health, focusing on their effects on intestinal microecology, their association with intestinal oxidative stress and immunity, and their implications for intestinal diseases. Our comprehensive evaluation of existing literature reveals that food additives play a significant role in intestinal health, with both potential risks and benefits. Certain additives May adversely affect intestinal health by disrupting the intestinal barrier, altering the balance of intestinal microecology, and promoting oxidative stress and inflammation. Conversely, some additives May have positive effects under specific conditions, such as providing antioxidant protection or promoting the growth of beneficial intestinal microorganisms. These findings underscore the need for individualized assessment of the safety and benefits of food additives, considering both their potential risks and advantages.

Looking ahead, we propose several directions for future research and action. First, there is a need to improve risk assessment methods, developing more accurate models and techniques to evaluate the safety of food additives under real-life use scenarios. This includes utilizing high-throughput screening techniques, advanced *in vitro* and *in vivo* modeling, and computational simulations to predict the long-term effects of food additive intake on human health. Second, we encourage the development of new, safer additives, particularly natural food additives with lower toxicity and better health benefits. Special emphasis should be placed on additives that support intestinal health, such as prebiotics and natural antioxidants with anti-inflammatory properties. Third, regulatory policies should be continually updated to reflect emerging evidence on the effects of food additives, ensuring effective protection of public health. This includes raising public awareness about food additive safety. We recommend that consumers carefully read food labels and prioritize products with safe, natural additives. Researchers should conduct in-depth clinical and population studies to verify laboratory findings and develop safe alternatives. Policymakers should base regulatory decisions on the latest scientific evidence and promote international cooperation in food safety standards. Through these concerted efforts, we can enhance our understanding of food additives’ impact on intestinal health, develop safer and more effective additives, and ultimately achieve both food safety and public health goals.
